# Design Optimization and *In Vitro*-*In Vivo* Evaluation of Orally Dissolving Strips of Clobazam

**DOI:** 10.1155/2014/392783

**Published:** 2014-09-28

**Authors:** Rajni Bala, Sushil Khanna, Pravin Pawar

**Affiliations:** ^1^Chitkara College of Pharmacy, Chandigarh-Patiala National Highway, Rajpura, Patiala, Punjab 140 401, India; ^2^Crest Healthcare Pvt. Ltd., Baddi, Himachal Pradesh 173205, India

## Abstract

Clobazam orally dissolving strips were prepared by solvent casting method. A full 3^2^ factorial design was applied for optimization using different concentration of film forming polymer and disintegrating agent as independent variable and disintegration time, % cumulative drug release, and tensile strength as dependent variable. In addition the prepared films were also evaluated for surface pH, folding endurance, and content uniformity. The optimized film formulation showing the maximum *in vitro* drug release, satisfactory *in vitro* disintegration time, and tensile strength was selected for bioavailability study and compared with a reference marketed product (frisium5 tablets) in rabbits. Formulation (F6) was selected by the Design-expert software which exhibited DT (24 sec), TS (2.85 N/cm^2^), and *in vitro* drug release (96.6%). Statistical evaluation revealed no significant difference between the bioavailability parameters of the test film (F6) and the reference product. The mean ratio values (test/reference) of *C*_max_ (95.87%), *t*_max_ (71.42%), AUC_0−*t*_ (98.125%), and AUC_0−∞_ (99.213%) indicated that the two formulae exhibited comparable plasma level-time profiles.

## 1. Introduction

Oral route is one of the most preferred routes of drug administration due to its safety, ease of administration, and acceptability by patients. About 60% of conventional dosage forms are available as the oral solid dosage forms [1]. The low bioavailability, longer onset of action, and dysphasia patients turned the manufacturer towards the parenterals and liquid dosage forms. But the liquid dosage forms (syrup, suspension, emulsion, etc.) have the problem of accurate dosing and parenterals are painful drug delivery systems, so they result in patient incompliance. The most popular oral dosage forms are tablets and capsules; one major drawback of these dosage forms is the difficulty to swallow [2]. Drinking water plays an important role in the swallowing of oral dosage forms. People experience inconvenience in swallowing tablet dosage forms when water is not available particularly in the case of traveling (motion sickness) and sudden episodes of coughing during the common cold, allergic condition, and bronchitis. Under such circumstances, tablets that can rapidly dissolve or disintegrate in the oral cavity known as fast dissolving tablets have attracted a great deal of attention. Fast dissolving tablets are also known as mouth-dissolving tablets, orodispersible tablets, rapidmelts, and porous tablets. Fast dissolving tablets dissolve or disintegrate within 60 seconds when placed in the mouth without drinking water or chewing. The active ingredients are absorbed through mucous membranes in the mouth and GIT and enter the blood stream [3]. But due to certain disadvantages like their physical solid form, psychological fear of swallowing, chewing, or chocking, friability of wafer like porous and low pressure moulded tablet, and expensive packaging cost of these dosage forms to protect them, a new technology was developed as orally dissolving strip. Orally dissolving strips are the most advanced form of oral solid dosage form due to more flexibility and comfort [4]. It improves the efficacy of APIs by dissolving within a minute in oral cavity after the contact with saliva without chewing and need of water for administration. It gives quick absorption and instant bioavailability of drugs due to high blood flow and permeability of oral mucosa which is 4–1000 times greater than that of skin. Orally dissolving strips are useful in patients such as pediatrics, geriatrics, bedridden, and emetic patients and conditions such as sudden episodes of allergic attacks or coughing. They can be used for local and systemic delivery. There is an increasing interest in the development of orally dissolving strips as an alternative to fast dissolving tablets [5], due to their faster dissolution rate, higher flexibility, and better patient compliance. Presently, research work on the use of orally dissolving strips as promising carriers for the delivery of multiple active pharmaceutical ingredients has emerged [6–11]. Marketed orally dissolving strips products have also become available including Listerine, Chloraseptic, Triaminic, and multivitamins [12]. The backbone of an orally dissolving strip is generally formed of a plasticizer and film forming polymer or a mixture of polymers that provide the necessary elasticity and shape to the film. Examples of polymers that have been used in the formulation of orally dissolving strips include hydrocolloids or povidone K-90 [13], maltodextrin (MDX), hydroxypropyl ethylcellulose (HPMC-E15, 5), pectin, sodium alginate [14], or blends of polymers [15]. Orally dissolving strips can be prepared using a solvent-casting, rolling, hot melt extrusion, or solid dispersion methods. Epilepsy is a condition characterized by the repeated attacks of epileptic seizures. Epileptic seizures can occur in nonepileptic patients subjected to a variety of stresses and stimuli. Epilepsy is a neurological disorder which requires quick management of seizures in order to avoid the risk of permanent brain damage [16]. Pharmacotherapy with antiepileptic drugs remains the major treatment modality for epilepsy. Management of epilepsy differs from the treatment of other diseased conditions in that a single epileptic attack has a major negative effect on quality of life. Clobazam is a newer 1, 5-benzodiazepine derivative which is a well tolerated, safe, and very effective antiepileptic drug having a broad spectrum of antiepileptic activity and minimal side effects and being relatively inexpensive. Its wider use is recommended in children with intractable epilepsy [17]. Thus, to control the epileptic seizures in the shortest possible time, an attempt has been made to develop, evaluate, and optimize orally dissolving strips of clobazam with improved bioavailability and palatability. Clobazam is an ideal drug candidate for an orally dissolving strip formulation because of its indication in children and its low-dose requirement. The formulation of clobazam as an orally dissolving strip, required to be placed on the patient's tongue without swallowing for dose administration, would significantly facilitate dose administration, with subsequent improvement in patient compliance. Thus, the aim of this work was to design, characterize, and optimize orally dissolving strip of clobazam using two polymers: SSG (disintegrant) and PVA (film former). A 3^2^ factorial design was used to evaluate the influence of film forming polymer PVA and disintegrating agent SSG on the film's mechanical properties, disintegration time, and dissolution rate. This study also assessed the* in vivo* performance and IVIVC of the optimum formulation by administration to healthy rabbits.

## 2. Material and Method

### 2.1. Material

Clobazam was received as a gift sample from Consern Pharma Pvt. Ltd., Ludhiana, India. Sodium starch glycolate, PEG-400, and directly compressible mannitol were received from Loba chemie Pvt. Ltd., Mumbai, India. Polyvinyl alcohol was procured from Central Drug House Pvt. Ltd., New Delhi, India. All other chemicals used were of analytical reagent grade.

### 2.2. Fourier Transform Infrared (FTIR) Analysis

The pure drug clobazam and physical mixture of clobazam and polymers were mixed with IR grade KBR pellets in the ratio of 100 : 1 and corresponding pellets were prepared in a hydraulic press. The pellets were scanned over a wave number range of 4000–500 cm^−1^ in using Perkin Emler spectrum 400USA, FTIR instrument.

### 2.3. Differential Scanning Calorimetric (DSC) Analysis

Differential scanning calorimetry (DSC) analysis was undertaken to visualize the changes, if any, observed during the preparation of the orally dissolving strip using Mettler Toledo model DSC 821e instrument. DSC of clobazam (pure drug), physical blend of PVA, clobazam and SSG (polymer), and optimized film formulation F6 were carried out over a temperature range of 30 to 300°C at a scanning rate of 5°C/min.

### 2.4. Solubility Studies of Pure Clobazam in Phosphate Buffer pH 6.8 and Different Solvents

Solubility studies were carried out by taking [18] an excess amount of drug in 10 mL of different solvents and pH 6.8 buffers in conical flask, closed with aluminum foil and constantly agitated at room temperature for 24 hrs, using orbital shaking incubator (Remi Instruments, C-24 BL, and Mumbai, India). Further, the solutions were filtered and the amount of drug solubilised was estimated at a wave length of 232 nm by using Systronics PC based double beam spectrophotometer 2202, Mumbai, India.

### 2.5. Preparation of Orally Dissolving Strips

The orally dissolving strips of clobazam using film forming polyvinyl acetate (PVA) and PEG 400 as plasticizer were prepared by solvent-casting method [19]. An aqueous solution of the polymer was prepared in warm distilled water and was kept aside for 4 hours for swelling of polymer. Clobazam was added to the aqueous polymeric solution after levigation with required volume of PEG 400. This was followed by the addition of mannitol as a sweetener as well as a solubilizer and sodium starch glycolate as a superdisintegrant. The solution was casted on a plastic Petri dish and dried at room temperature for 24 hr. The strip was carefully removed from the Petri dish, checked for any imperfections, and cut into the required size (2 × 2 cm^2^) to deliver the equivalent dose of 5 mg per strip. The film samples were stored in desiccators for further analysis. Preliminary trials were undertaken for designing the orally dissolving strips where the effect of various concentrations of the film forming agent and superdisintegrants on the characteristics of the strips was noted. In addition, the prepared strips were also checked for surface perfection, smoothness, and ease of removal from Petri dish without rupturing, folding, or cracking.

### 2.6. Preparation of Orally Dissolving Strips of Clobazam Using 3^2^ Factorial Designs

A 3^2^ full factorial design was employed to study the effect of independent variables *X*_1_ (PVA) and *X*_2_ (SSG) over the dependent variables like tensile strength (N/cm^2^), disintegration time (sec), and* in vitro* drug release (%) as shown in design layout Tables 1 and 2. In this design, two factors were evaluated each at three levels (−1, 0, +1) and all possible nine experimental batches were formulated. Composition of all nine possible combinations of orally dissolving strip of clobazam using 3^2^ full factorial designs is shown in Table 3. The data was subjected to contour and 3D response surface plot using Design-expert software version 8.0.7.1. A multiple linear regression equation incorporating interactive and polynomial terms was used to calculate the response as follows:

(1) $$\begin{array}{c} Y=b_{0}+b_{1}X_{1}+b_{2}X_{2}+b_{12}X_{1}X_{2}+b_{11}X_{1}^{2}+b_{22}X_{2}^{2}, \end{array}$$

where *Y* is the dependent, that is, response variable, namely, disintegration time, tensile strength, and* in vitro* drug release; *b*_0_ is the arithmetic mean response of the nine runs; and *b*_1_ and *b*_2_ are the estimated coefficients for the factors *X*_1_ and *X*_2_, respectively. The main effects (*X*_1_ and *X*_2_) represent the average result of changing one factor at a time from its low to high value. The interaction term (*X*_1_ *X*_2_) shows how the response changes when two factors are simultaneously changed. The polynomial terms (*X*_1_^2^ and *X*_2_^2^) are used to check nonlinearity. The polynomial equations can be used to draw conclusions after considering the magnitude of the coefficient and the mathematical sign it carries (i.e., positive or negative). The high values of the correlation coefficients for the dependent variables indicate a good fit.

### 2.7. Physicochemical Characterization of Clobazam Orally Dissolving Strips

#### 2.7.1. Film Weight and Thickness

The thickness of each strip was measured at five different locations (centre and four corners) using calibrated digital Vernier caliper (Mituotoyo, Japan). Data are represented as a mean ± SD of three replicate determinations.

#### 2.7.2. Folding Endurance

The folding endurance was measured manually for the prepared strips. A strip of (2 × 2 cm^2^) was cut and repeatedly folded at the same place till it broke. The number of times the strip could be folded at the same place without breaking was taken as a measure of folding endurance.

#### 2.7.3. Drug Content Determination

The oral strip of size 4 cm^2^ was dissolved in 10 mL of phosphate buffer pH 6.8 and solution was filtered and drug content was estimated at 232 nm using double beam UV/Visible spectrophotometer (Systronics 2202, Mumbai, India). The experiments were carried out in triplicate for the strips of all formulations and average values were recorded [20].

#### 2.7.4. Surface pH

The surface pH of orally dissolving strips was determined in order to investigate the possibility of any side effects* in vivo*. As an acidic or alkaline pH may cause irritation to the oral mucosa, it is determined to keep the surface pH as close to neutral as possible. Strip was wetted with the help of water. The pH was measured by bringing the electrode in contact with the surface of the oral strip. This study was performed on three strips of each formulation and mean ± SD calculated [21].

#### 2.7.5. *In Vitro* Disintegration Time of Orally Dissolving Strip of Clobazam

*In vitro* disintegration time was measured by placing the film (2 × 2 cm^2^) on stainless steel wire mesh placed in a Petri dish containing 10 mL of phosphate buffer pH 6.8. Time required for the oral strip to break was noted as* in vitro* disintegration time. The test was performed on three strips of each formulation batch and mean ± SD was calculated [22].

#### 2.7.6. *In Vitro* Dissolution Studies of Clobazam Orally Dissolving Strip

*In vitro* dissolution test was performed according to the USP type II paddle apparatus (Labindia DS 8000, Mumbai, India). Test was performed by fixing the oral strip (2 × 2 cm^2^) to rectangular glass plates so as to prevent it from floating and it was placed at the bottom of dissolution vessel containing 900 mL of phosphate buffer pH 6.8 at 37°C with a rotation speed of 50 rpm. A 5 mL of sample was taken at time intervals from 1 to 30 min, and the same volume was replenished with fresh buffer solution maintained at 37°C. The samples were filtered and analyzed at 232 nm using double beam UV/Visible spectrophotometer (Systronics 2202, Mumbai, India); the content of drug was calculated using equation generated from standard calibration curve of clobazam. The release mechanism of clobazam from oral strip was also determined by fitting the release data to different kinetic models, zero order [23], first order [24], and Higuchi [25].

#### 2.7.7. Surface Morphology

The surface morphology of the optimized orally dissolving strip formulation was observed with scanning electron microscope (Hitachi S-3400N type II model, Japan). Pictures were taken at an excitation voltage of 1.0 KV and a magnification of 1000x.

#### 2.7.8. Determination of Moisture Uptake

Films were cut into 2 × 2 cm square strips (4 cm^2^). The moisture uptake by the strip (*n* = 3) was determined by exposing them to an environment of 75% relative humidity (RH) at room temperature (25 ± 2°C) for 1 week [26, 27]. The uptake of moisture by the strips was measured and calculated as percent increase in weight.

#### 2.7.9. Tensile Strength of Orally Dissolving Strips of Clobazam

Tensile strength testing was determined at Central Institute of Post Harvesting Engineering and Technology (CIPHET), Ludhiana, India, using a texture analyzer TAHDi (stable microsystem), equipped with a 5 kg load cell. The strip was cut into 100 × 12.5 mm strips and equilibrated at 25°C for 1 week. Each test strip was longitudinally placed in the tensile grips on the texture analyzer. Initial grip separation was 60 mm and crosshead speed was 50 mm min^−1^. The test was considered concluded at the point where the oral strip breaks. Tensile strength, elongation at break, was computed to evaluate the tensile properties of the strips. Tensile strength (TS) was calculated by dividing the maximum load by the original cross-sectional area of the strip and it was expressed as (N/cm^2^). Percent elongation at break (*E*%) was calculated by dividing the length at the time of break of the strip by the initial length of the strip and multiplying by 100 using the following equation:

(2) $$\begin{array}{c} E\%=\frac{L-L_{0}}{L_{0}}\times 100, \end{array}$$

where *L*_0_ is the initial length of the strip and *L* is the length at the time of break. An average of three measurements was taken for each formulation [28].

### 2.8. HPLC Method Validation Used to Quantify Clobazam in Rabbit Plasma

The calibration curve was performed with standards of the final concentrations of 5, 10, 25, 50, and 100 ng/mL in rabbit plasma [29]. The intraday, interday precisions and accuracy of the method were determined with three replicates spiked plasma samples at different concentrations of clobazam. The intraday and interday variation was calculated in terms of percent relative to standard deviation.

### 2.9. Pharmacokinetic Study of Selected Clobazam Orally Dissolving Strip Formulation

The study was conducted in accordance with the principles of laboratory animal care. Six rabbits of either sex (weighed 2.5 ± 0.2 kg) were selected for the study. All the rabbits were healthy during the period of study. All the rabbits were fasted overnight before the administration of the selected fast dissolving film and marketed formulation but had free access to water. The rabbits were randomly divided into two groups with each group containing three rabbits (*n* = 3). The rabbits were positioned on a table with lower jaw supported in a horizontal position and orally dissolving strip was carefully placed on the rabbit tongue in one group as shown in Figure 12. The marketed tablet was administered orally to another group. Blood samples for pharmacokinetic analysis were collected from marginal ear veins of rabbits immediately before drug administration and at 5, 10, 15, 30, 60, 120, 180 min, 6 hrs, 12, and 24 hrs interval. The plasma samples were prepared by mixing 0.1 mL of plasma with 0.05 mL ibuprofen as internal standard (from the stock 6000 ng/mL) in a clean Eppendorf polypropylene tube and then extracting with 1.5 mL of acetonitrile after vertical agitation (1 min) and centrifugation at 1000 rpm, for 5 min. The upper organic layer was injected onto the HPLC system for analysis. The HPLC analysis was performed using Agilent 1200 series system by using a mobile phase composed of water pH 3.5, adjusted with orthophosphoric acid and acetonitrile (55 : 45 v/v), and binary eluted at a flow rate of 1.5 mL min^−1^ [30]. Protocols for all the animal studies were approved by institutional ethical committee* [IAEC/CCP/12/PR-007]*.

### 2.10. Calculation of Pharmacokinetic Parameters

Plasma concentration time profile curve of clobazam was plotted. Maximum plasma concentration *C*_max⁡_ and the time *t*_max⁡_ were obtained directly from the individual plasma concentration versus time curves. The terminal half-life, *t*_1/2_, was obtained from log linear regression analysis of the plasma concentration time curves in the terminal phase. The area under plasma concentration time curve (AUC_0−*t*_, and AUC_0−*∞*_) was determined by linear trapezoidal method. For 90% confidence interval, the AUC and *C*_max⁡_ values were transformed into their respective logarithms and analysis of variance was calculated using software Kinetica 5.0. Trial version Adept Scientific limited.

### 2.11. IVIV Correlation

*In vitro* and* in vivo* correlations were carried out to compare the release of drug. Here, the* in vivo* percentage of the drug absorbed was plotted against the* in vitro* percentage of the drug released to determine the correlation coefficient. The fraction of the drug absorbed was determined using the Wagner Nelson method, using the following equation [31]:

(3) $$\begin{array}{c} F_{a}=\left[\frac{\left(C_{t}+k_{e}{\text{AUC}}_{0-t}\right)}{k_{e}{\text{AUC}}_{0-\infty }}\right]\times 100, \end{array}$$

where *F*_*a*_ is the fraction of drug absorbed, *C*_*t*_ is the plasma drug concentration at time *t*, *k*_*e*_ is the overall elimination rate constant, and AUC_0−*t*_ and AUC_0−*∞*_ are areas under the curve between time zero and time *t* and between time zero and infinity, respectively.

### 2.12. Stability Studies

The optimized orally dissolving formulation of clobazam was subjected to stability studies by packing the individual strip in aluminum foil and loading the formulation on Remi stability chamber SC-10 Plus (Remi elektrotechnik Ltd., Vasai, India) as per ICH guidelines for 180 days at 40°C ± 2°C/75% RH ± 5%. Samples were withdrawn at regular intervals and evaluated for* in vitro* drug release, disintegration time, and tensile strength.

## 3. Results and Discussion

### 3.1. Fourier Transform Infrared (FTIR) Analysis

**Figure 1:**
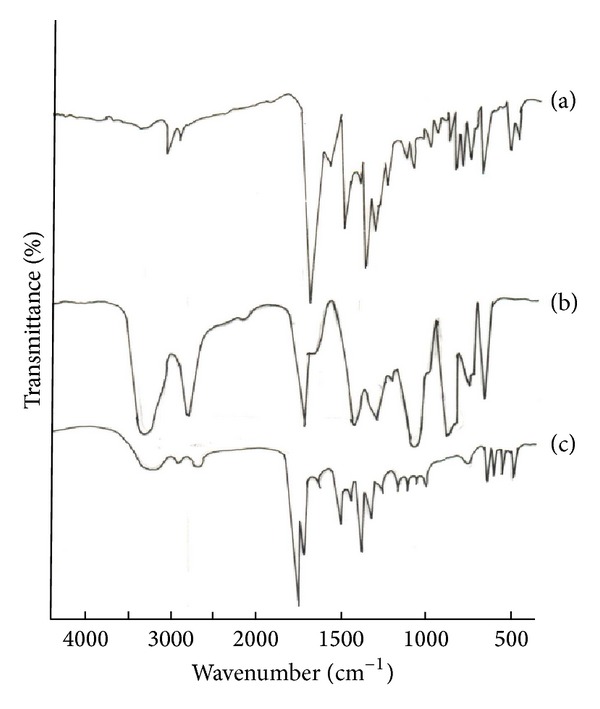
FTIR of (a) clobazam, (b) PVA, and (c) physical mixture of clobazam and PVA.

FTIR spectra of pure drug and physical mixture of PVA and clobazam as shown in Figures 1(a), 1(b), and 1(c) indicate that there was no interaction between drug and film forming polymer used. Pure clobazam displays a peak characteristic of C=O stretching vibration at 1694.7 cm^−1^, aromatic CH stretching at 3075.4 cm^−1^, C–C stretching at 1493.5 cm^−1^, C–N stretching at 1100–1200 cm^−1^, CH bending at 600–800 cm^−1^, and CH_3_ bending at 1371 cm^−1^; the spectra of drug with PVA showed all characteristics peaks of drug indicating that the drug is compatible with PVA.

### 3.2. Differential Scanning Thermograms

**Figure 2:**
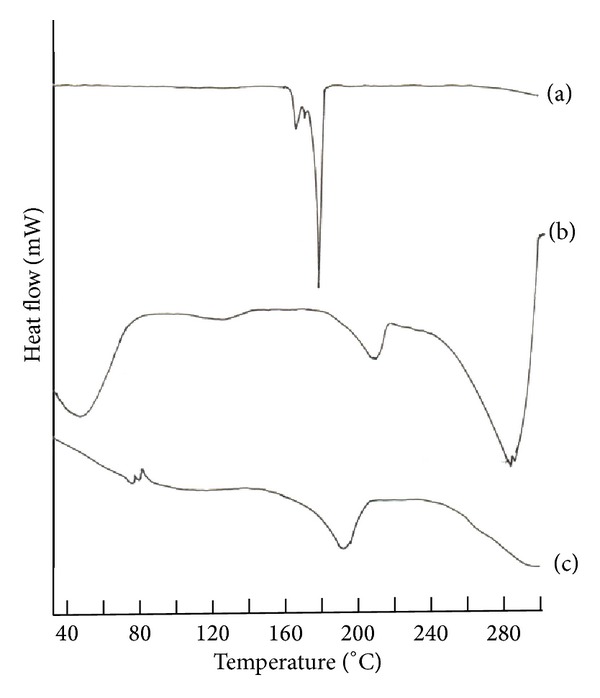
DSC thermogram of (a) clobazam, (b) F6, and (c) physical blend of clobazam, PVA, and SSG.

DSC thermograms of clobazam (pure drug), PVA (polymer), clobazam and SSG, and clobazam loaded orally dissolving strip as shown in Figures 2(a), 2(b), and 2(c) illustrated a sharp endothermic peak corresponding to the melting of crystalline clobazam at 180°C. The melting endothermic peak of clobazam was not observed in the drug loaded PVA orally dissolving strip. This indicates that clobazam was uniformly dispersed and present in an amorphous state in the polymeric matrix.

### 3.3. Physicochemical Characterization of Clobazam Orally Dissolving Strip

Physicochemical characterization of clobazam films as given in Table 4 indicated that all the strips prepared with different polymer concentrations were flexible, smooth, transparent, nonsticky, and homogeneous. The thickness of the oral strip varies from 0.140 to 0.29 mm. It was observed that there was no significant difference in the thickness among the strips, which indicated that the orally dissolving strips were uniform. The weight of oral strips varies from 43.0 to 63.0 mg. The folding endurance of the oral strips varies from 150 to 284. F6 exhibited good folding endurance, indicating good flexibility. The folding endurance increases with the increasing content of PVA. However, the folding endurance of the oral strip F7–F9 was found to decrease with increasing content of PVA. Thus, it appears that the increasing amount of PVA decreases the flexibility of the strip. Since the surface pH of orally dissolving strips was found to be around neutral pH, there will not be any kind of irritation to the mucosal lining of the oral cavity. All formulations were found to contain uniform quantities of drug ranging between 97.93% and 99.96%, as indicated by content uniformity studies.

### 3.4. *In Vitro* Disintegration Time of the Orally Dissolving Strips of Clobazam

*In vitro* disintegration time of the formulated orally dissolving strips was found to decrease with the addition of SSG. Increase in the concentration of superdisintegrant decreases the disintegration time of films, which was observed in F1, F2, F3, and F4; as the amount of PVA increases, the disintegration time increases because of the increasing thickness of the strip but again decreases with increasing amount of SSG and formulation with F6 exhibited minimum disintegration time. Mathematical relationship generated using multiple linear regression analysis for the studied variable is expressed as follows:

(4) disintegration  time=28.32222+1.56667X1 −4.33333X2−0.075000X1X2 −0.33333X12+1.66667X22.

The Model* F*-value of 42.62 implies that the model is significant.

### 3.5. *In Vitro* Dissolution Time

*In vitro* drug release was found to decrease with increase in the film forming polymer concentration which may be due to increase in the thickness of the oral strip and due to increase in the time required for wetting and dissolving the drug molecule present in the polymeric matrix but increase with increase in the concentration of the disintegrant. Formulations F1, F2, and F3 showed drug release up to 79.1%, 85%, and 89%, respectively, as shown in Figures 4(a), 4(b), and 4(c) at the end of 30 min. Film formed with higher quantity of polymer had shown slower dissolution rate; this might be due to the increase level of PVA that results in formation of high viscosity gel layer due to more intimate contact between the particles of polymer resulting in decrease in the mobility of drug particles from the swollen matrix, which leads to a decrease in the release rate [32]. The order of drug release in each set of formulation can be given as

(5) $$\begin{array}{c} \text{F}1<\text{F}2<\text{F}3,\\ \text{F}4<\text{F}5<\text{F}6,\\ \text{F}7<\text{F}8<\text{F}9. \end{array}$$

The formulation F6 showed a maximum percentage drug release of 96.6% in 30 min as shown in Figure 4(b). Reflection point at 30 min in the curve might be due to the complete solubilization of PVA in the medium which improve the wettability and solubilize the drug and provide the fast dissolution rate. Further, drop in the percent of drug release after 30 min might be due to the availability of less number of drug molecules in the free void spaces and a decrease in mobility through polymeric matrix. Mathematical relationship generated using multiple linear regression analysis for the response variable is expressed as follows:

(6) % drug  release=81.70111−5.16833X1 +5.1683X2+1.00250X1X2 −4.5016X12  +1.99833X22.

The Model* F*-value of 77.23 implies that the model is significant.

### 3.6. Tensile Strength of the Orally Dissolving Strips of Clobazam

Tensile strength and % elongation were found to increase with the increasing content of PVA which may be due the increase in the elasticity nature of the film forming polymer [33]. Mathematical relationship generated using multiple linear regression analysis for the studied variable is expressed as follows:

(7) Tensile  strength=2.39111+0.56500X1 +0.31167X2−0.01500X1X2 −0.1916X12+0.12833X22.

The Model* F*-value of 185.86 implies that the model is significant.

**Figure 3:**
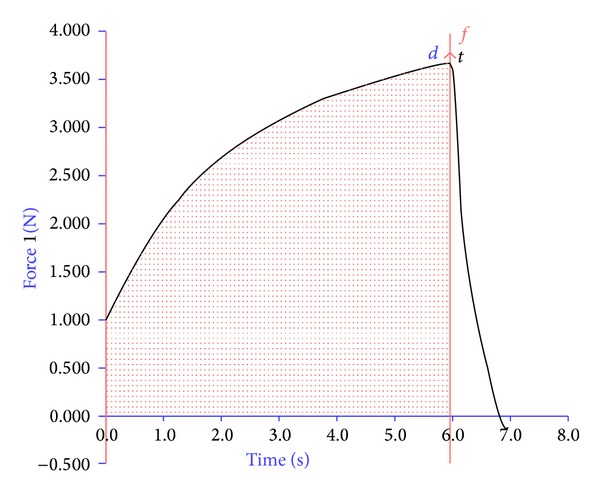
Strain-stress curves of the clobazam optimized ODF F6.

**Figure 4:**
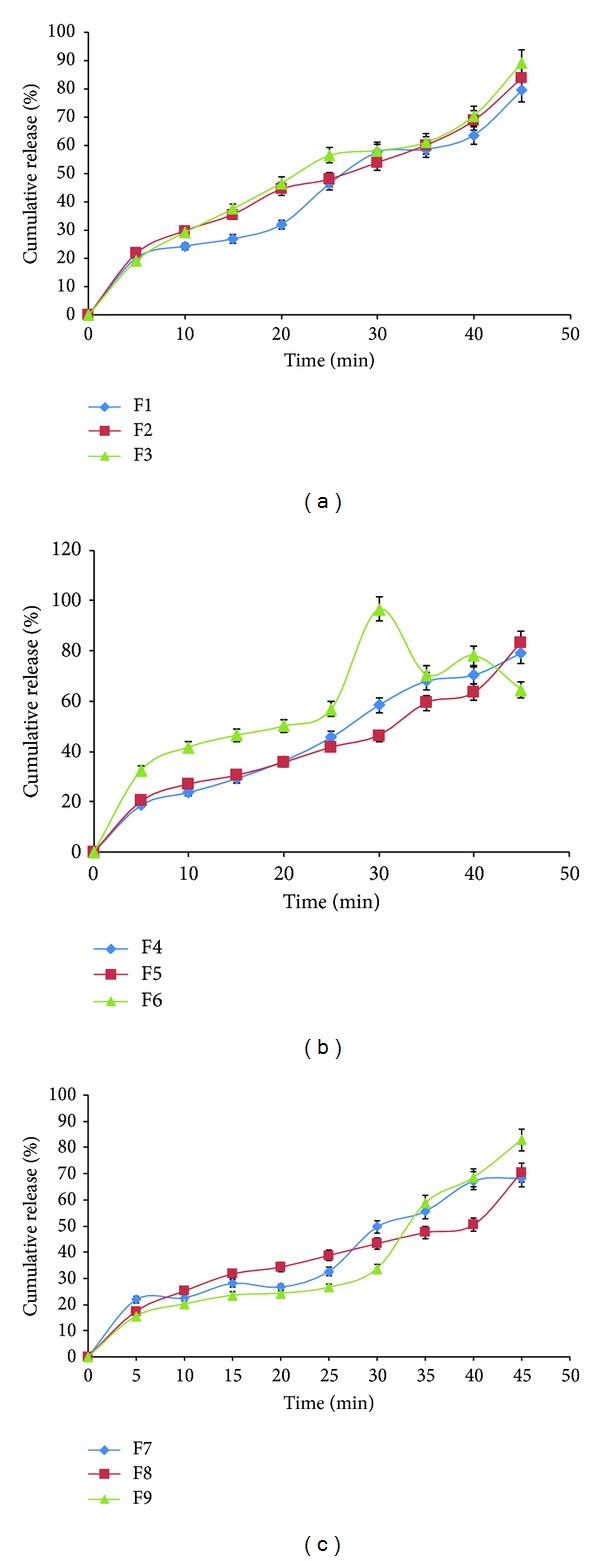
Clobazam release profiles from ODF formulations containing different levels of PVA as film former and SSG as superdisintegrant, (a) F1, F2, and F3, (b) F5, F6, and F7, and (c) F7, F8, and F9. Mean ± SD, *n* = 3.

Addition of SSG affects tensile strength and % elongation; higher concentration of SSG increases thickness and crystallinity of the film, which causes decrease in the tensile strength and % elongation. The stress strain curve of optimized ODF formulation F6 is shown in Figure 3.

### 3.7. Release Kinetics

There was a good linear correlation (*R* ^2^ = 0.9896 − 0.9999) obtained by plotting the percent of clobazam released from all the orally dissolving strip formulations against the square root of time. Thus, it was concluded that the release of clobazam from orally dissolving strip formulations followed a diffusion-controlled drug release profile and is in agreement with the Higuchi model. Results of release kinetics are shown in Table 5.

### 3.8. Regresion Analysis and Optimization

**Figure 5:**
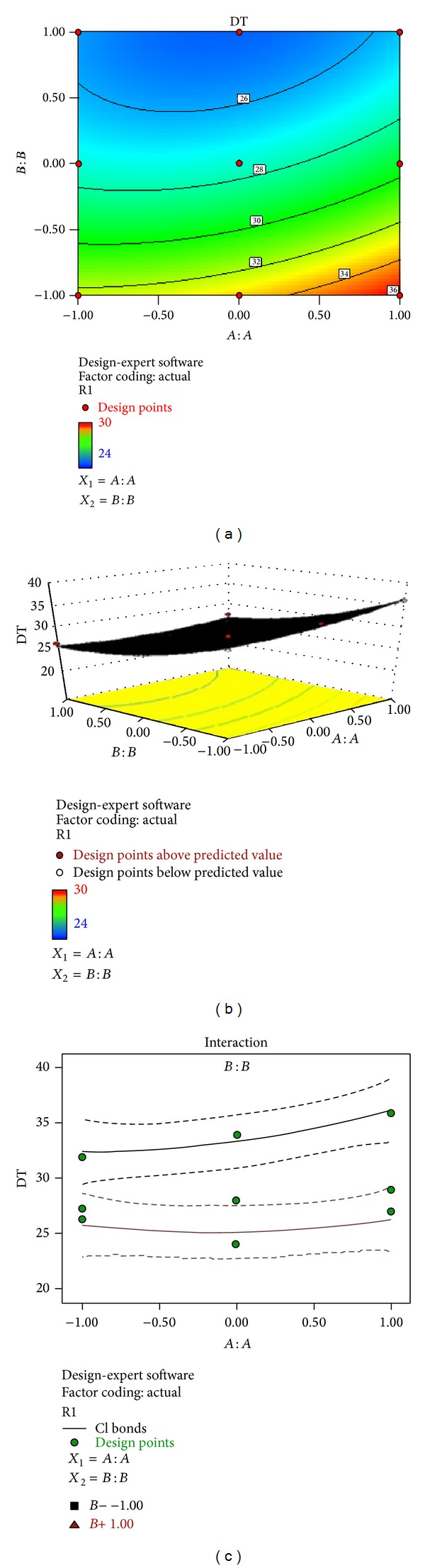
(a) Contour plot showing the relationship between various levels of 2 independent variables. (b) Response surface plot showing the influence of film forming polymer (PVA) and superdisintegrant (SSG) over disintegration time. (c) Corresponding plot showing the interaction between two factors.

**Figure 6:**
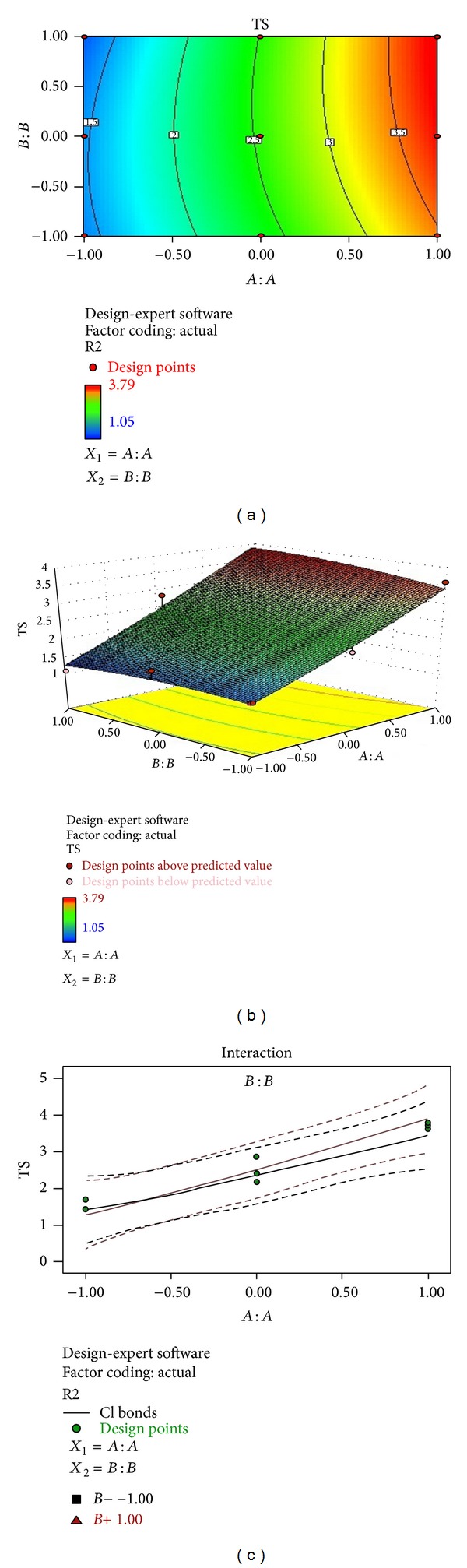
(a) Contour plot showing the relationship between various levels of 2 independent variables. (b) Response surface plot showing the influence of film forming polymer (PVA) and superdisintegrant (SSG) over tensile strength. (c) Corresponding plot showing the interaction between two factors.

**Figure 7:**
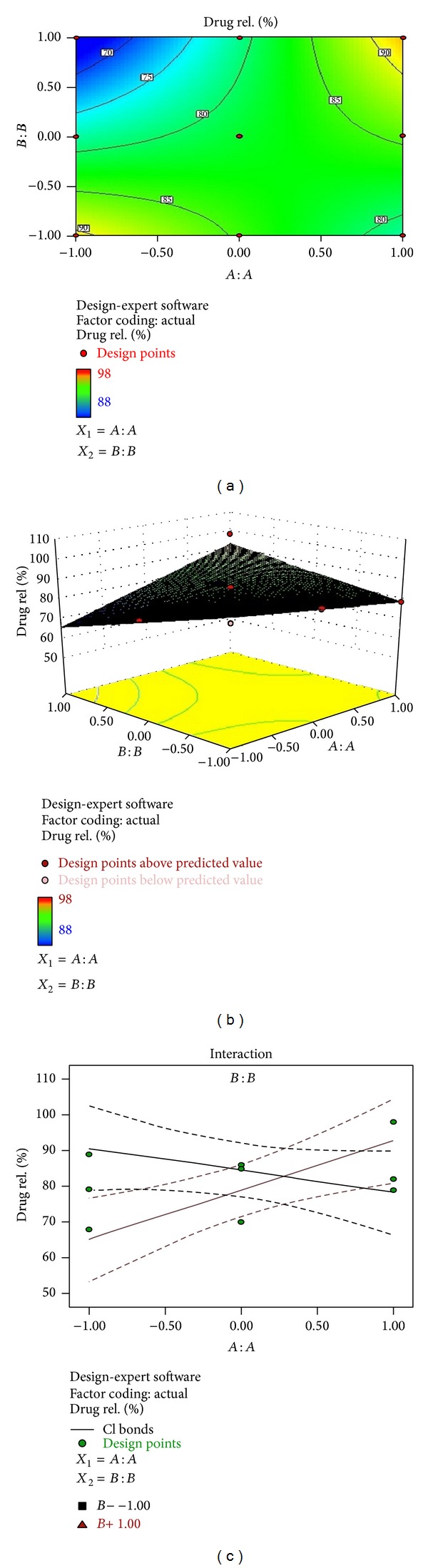
(a) Contour plot showing the relationship between various levels of 2 independent variables. (b) Response surface plot showing the influence of film forming polymer (PVA) and superdisintegrant (SSG) over % drug release. (c) Corresponding plot showing the interaction between two factors.

The results of multiple linear regression analysis are shown in Table 6, indicating that for all response variables which are disintegration time, tensile strength, and % drug release, the amount of PVA (*X*_1_) had a negative effect while the concentration of SSG (*X*_2_) had a positive effect; it means that as the amount of SSG increases the tensile strength, disintegration time decreases and there is increase in % drug release, while as the amount of PVA is increased, both the tensile strength and disintegration time increase [34–36]. Therefore, high level of SSG and medium level of PVA should be selected for the rapid disintegration and a faster* in vitro* drug release of the film. The observed values are in good agreement with the predicted values for the optimized formulation, which demonstrate the feasibility of surface response method in the formulation of oral fast dissolving films. The comparison of observed values and predicted values with % prediction error is shown in Table 7. The data of the response surface plot as shown in Figures 5, 6, and 7 demonstrated that both *X*_1_ and *X*_2_ affect the disintegration time, tensile strength, and % release of drug.

### 3.9. Scanning Electron Microscopy (SEM)

**Figure 8:**
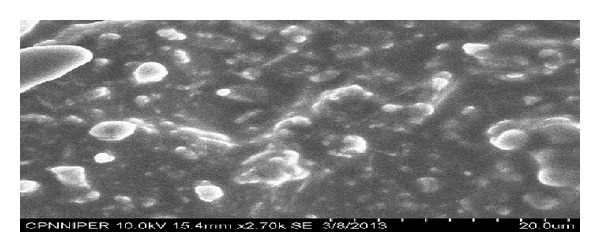
Surface morphology of optimized fast dissolving film formulation of F6.

The surface morphology as shown in Figure 8 using scanning electron microscopy of the optimized orally dissolving strip formulation of clobazam depicted smooth surface with some little pores, which is an indication of uniform distribution of drug particles.

### 3.10. Comparison of the Optimized Formulation with Marketed Formulation

**Figure 9:**
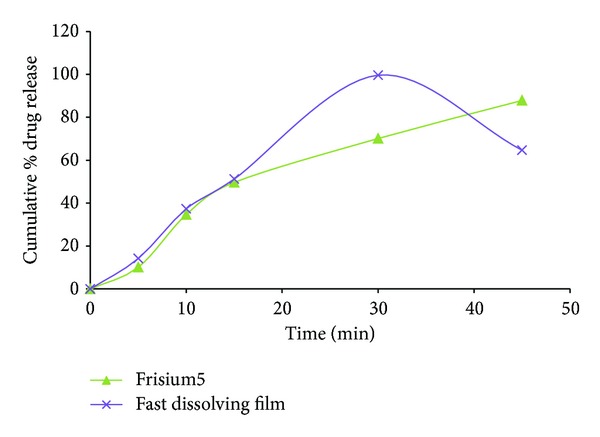
*In vitro* release profiles of clobazam from optimized ODF formulation F6 and frisium5. Mean ±  *n* = 3.

Based on the responses the formulation F6 showing the highest dissolution rate,* in vitro* disintegration time suitable for fast-dissolving dosage form, and satisfactory tensile strength properties was chosen for subsequent comparative study relative to a marketed clobazam formulation as shown in Table 8. Comparative results of* in vitro* drug release of F6 formulation and marketed formulation (frisium5) as shown in Figure 9 indicate that the optimized formulation was comparable to the marketed formulation.

### 3.11. HPLC Method Validation Used to Quantify Clobazam in Rabbit Plasma

The results from the HPLC method validation in the rabbit plasma are shown in Table 9. The within day and between day precision as given in terms of % relative standard deviation ranged from 0.5 to 6.2% and from 1.1 to 1.6%, respectively.

### 3.12. Pharmacokinetic Parameters

**Figure 10:**
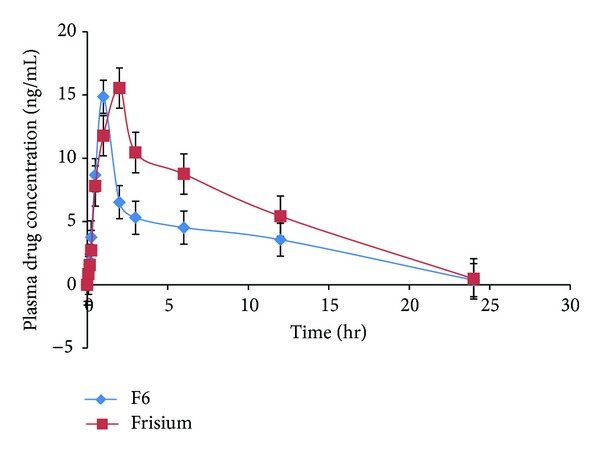
Means of plasma concentrations and time profiles of clobazam from (F6) ODF and marketed formulation (frisium5) mean ± SD *n* = 3.

The mean clobazam plasma concentration time profiles for the prepared orally dissolving strip and the marketed tablets are shown in Figure 10. The statistical comparison of *C*_max⁡_, AUC_0−*t*_, and AUC_0−*∞*_ indicated no significant difference between the two treatments (test and reference marketed tablets). The 90% confidence intervals for the mean ratio (test/reference) of *C*_max⁡_, AUC_0−*t*_, and AUC_0−*∞*_ were 95.87%, 98.12%, and 99.21%, respectively, and are shown in Table 10, while the acceptable range is 80–125% for AUC_0−*t*_ and AUC_0−*∞*_, and 70–143% for *C*_max⁡_, as proposed by the FDA [37].

### 3.13. IVIV Correlation

**Figure 11:**
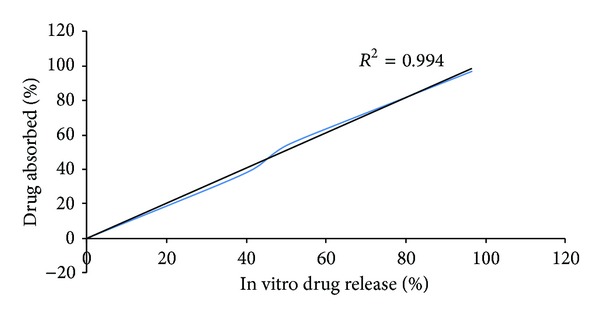
IVIVC plot for clobazam film formulation F6 in phosphate buffer pH 6.8.

**Figure 12:**
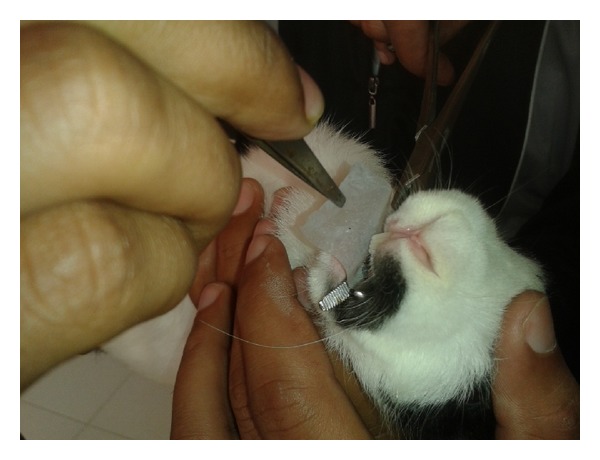
Bioavailability study on clobazam optimized ODF F6 and marketed formulation (frisium5).

*In vitro-in vivo* correlation as shown in Figure 11 of percentage drug absorbed and percentage drug released showed good* in vitro-in vivo* correlation for optimized film formulation F6 with *R* ^2^ value 0.994.

### 3.14. Stability Studies

The optimized formulation F6 was selected for the stability study. Stability data are shown in Table 11. It was concluded that there was no significant change observed in the weight of orally dissolving strip, tensile strength, and %* in vitro* drug release. The average clobazam content of formulation F6 after 6-month storage was 95.1 ± 0.17 mg (ranging from 91.88 to 96.80%). The appearance of the film after storage for 6 months remained unchanged. Therefore, the prepared formulation was stable up to 6 months at 40°C (75%RH).

## 4. Conclusion

The orally dissolving strips of clobazam prepared using PVA as film forming material and SSG as disintegrant by the solvent-casting method showed satisfactory drug dissolution and acceptable physicomechanical characteristics. 3^2^ factorial design was used for the optimization, amongst nine formulations prepared as per the design layout, which indicates that the film prepared using 100 mg of PVA and 6% of SSG (F6) showed the highest dissolution rate, suitable* in vitro* disintegration time, and satisfactory tensile strength and was selected as the optimized formulation.* In vivo* studies also indicated absence of significant difference between F6 and frisium5 marketed tablets and both exhibited comparable drug plasma level-time profiles. Accelerated stability studies results showed that prepared formulation was stable enough for the period of at least 6 months. Therefore, the present orodispersible film formulation containing clobazam considered is potentially useful for the treatment of epileptic attack where improved patient compliance and convenience are expected and can be used as an alternative to the fast dissolving tablet formulation.

## Figures and Tables

**Table 1:** Independent variables and their levels.

Variables	Low level (−1)	Medium level (0)	High level (+1)
*X*_1_ = amount of SSG (%)	2	4	6
*X*_2_ = amount of PVA (mg)	50	100	200

**Table 2:** Factorial design with disintegration time, tensile strength, and % drug release obtained.

Run	Factor *X*_1_	Factor *X*_2_	DT (sec)	TS (N/cm^2^)	% drug release
1	−1	−1	32 ± 2.0	1.43 ± 0.03	79.1
2	0	−1	27.3 ± 3.1	1.68 ± 0.01	85
3	+1	−1	26.3 ± 1.0	1.057 ± 0.02	89
4	−1	0	34 ± 2.0	2.18 ± 0.03	79.4
5	0	0	28 ± 1.0	2.38 ± 0.03	83.1
6	+1	0	24 ± 0.5	2.85 ± 0.03	96.6
7	−1	+1	36 ± 2.1	3.61 ± 0.05	68.9
8	0	+1	29 ± 1.0	3.73 ± 0.03	70
9	+1	+1	27 ± 2.3	3.79 ± 0.02	82.1

DT = disintegration time.

TS = tensile strength.

**Table 3:** Composition of different orally dissolving strips containing clobazam.

Ingredients	Formulation batches								
	F1	F2	F3	F4	F5	F6	F7	F8	F9
Clobazam (mg)	5	5	5	5	5	5	5	5	5
PVA (mg)	50	50	50	100	100	100	200	200	200
PEG 400 (mg)	0.3	0.3	0.3	0.3	0.3	0.3	0.3	0.3	0.3
SSG (mg)	2	4	6	2	4	6	2	4	6
D-Mannitol (mg)	25	25	25	25	25	25	25	25	25
Water (mL)	10	10	10	10	10	10	10	10	10

The concentration of the drug was 5 mg/4 cm^2^ of the film.

**Table 4:** Characterization of different orally dissolving strips of clobazam.

Formulae	% drug content	Thickness (mm)	%*E*	Folding endurance	P^H^
F1	96 ± 3.05	0.14 ± 0.05	10 ± 1.1	200 ± 3.54	6.8
F2	98.53 ± 0.74	0.21 ± 0.1	20 ± 1.2	210 ± 2.44	6.9
F3	98.49 ± 2.54	0.22 ± 0.06	25 ± 0.5	280 ± 2.53	6.6
F4	99.11 ± 0.64	0.26 ± 0.1	25 ± 1.44	283 ± 0.44	7.0
F5	97.73 ± 0.50	0.27 ± 0.2	50 ± 1.43	280 ± 4.12	7.0
F6	99.96 ± 4.17	0.29 ± 0.05	150 ± 1.01	284 ± 1.22	7.0
F7	98.54 ± 1.66	0.26 ± 0.05	50 ± 1.23	150 ± 0.44	6.8
F8	97.93 ± 4.22	0.27 ± 0.3	75 ± 1.44	200 ± 2.34	7.0
F9	98.41 ± 3.23	0.27 ± 0.1	80 ± 1.11	180 ± 2.33	7.0

%*E* = % elongation.

Values are expressed as mean ± SD; *n* = 3.

**Table 5:** Release kinetic study of orally dissolving strips of clobazam using different kinetic models.

Formulation code	Kinetic models				
	Zero order	First order	Higuchi	Korsmeyer	Best fit kinetic
	*r* ^2^	*r* ^2^	*r* ^2^	*r* ^2^	
F1	0.947	0.826	0.971	0.967	Higuchi
F2	0.924	0.961	0.996	0.984	Higuchi
F3	0.978	0.984	0.997	0.986	Higuchi
F4	0.965	0.950	0.989	0.952	Higuchi
F5	0.985	0.952	0.994	0.992	Higuchi
F6	0.952	0.861	0.998	0.993	Higuchi
F7	0.702	0.703	0.987	0.929	Higuchi
F8	0.959	0.933	0.996	0.980	Higuchi
F9	0.848	0.881	0.988	0.980	Higuchi

**Table 6:** Summary of results of regression analysis.

Coefficient	*b*_0_	*b*_1_	*b*_2_	*b*_12_	*b*_11_	*b*_22_	*R* ^2^
Disintegration time (sec)	28.32222	1.56667	−4.3333	−0.07500	−0.333	1.6667	0.9242
Tensile strength (N/cm^2^)	2.3911	−0.56500	0.31167	−0.01500	−0.1916	0.12833	0.9124
% drug release	81.70	−5.1683	5.1683	0.100250	−4.5016	1.99833	0.7620

*b*_0_, *b*_1_, *b*_2_, *b*_12_,  *b*_11_, and *b*_22_ represent regression coefficient of the independent variables (*X*_1_, *X*_2_).

**Table 7:** Comparison of observed and predicted values with % prediction error.

Variables	Predicted value	Observed value	% prediction error
Disintegration time (sec)	23.8	24	0.833%
% drug release	97.8	96.6	1.24%
Tensile strength (N/cm^2^)	2.90	2.85	−1.75%

**Table 8:** Comparison of the optimized formulation with marketed formulation (*n* = 3).

Formulation	Disintegration time (sec)	% drug release	Thickness (mm)
Frisium	65 ± 1.53	79.2 ± 0.01	3.80 ± 0.02
F6	24 ± 0.5	96.6 ± 0.05	0.29 ± 0.05

**Table 9:** Intraday and interday precision accuracy of clobazam determination in rabbit plasma (*n* = 3).

Clobazam	Intraday		Interday		Accuracy	
Concentration (ng/mL)	Area	%RSD	Area	%RSD	Quantity	Recovery
					Spiked (ng/mL)	
10	4.324	0.5	5.085	1.1	10	10.57
20	6.049	0.9	6.158	1.1	20	18.09
25	7.655	6.2	8.798	0.3	25	25.55
40	12.06	3.0	12.24	0.8	40	39.36
50	15.23	2.4	16.32	1.4	50	49.58
75	22.88	0.8	20.59	0.4	75	74.76
100	30.98	0.7	30.95	1.6	100	100.69

**Table 10:** Comparison of pharmacokinetic parameters following administration of 5 mg clobazam in orodispersible film (F6) and frisium tablets to six rabbits (*n* = 3).

Parameters	Orodispersible film (mean ± SD) (test)	Frisium (mean ± SD) (reference)	Test/reference ratio
*C*_max⁡_ (ng/mL)	14.86 ± 4.9	15.545 ± 5.1	95.87
AUC_0–*t*_ (ng hmL^−1^)	87.018 ± 19.2	88.68 ± 16.2	98.125
AUC_0–*∞*_ (ng hmL^−1^)	90.726 ± 15.3	91.445 ± 14.2	99.213
*t*_max⁡_ (h)	1.0 ± 0.5	1.4 ± 0.5	71.428
*t*_1/2_ (h)	42.52 ± 12.5	45.24 ± 10.8	93.987

**Table 11:** Stability studies of optimized (F6) orally dissolving strip formulation of clobazam stored at 40°C/75% RH.

Parameters	Time points (days)					
	0	15	30	45	60	90
Disintegration time (sec)	24 ± 0.5	22.5 ± 0.1	23 ± 0.2	22 ± 0.5	23 ± 0.4	23.2 ± 0.5
Tensile strength (N/cm^2^)	2.85 ± 0.03	2.80 ± 0.02	2.81 ± 0.03	2.85 ± 0.03	2.70 ± 0.05	2.80 ± 0.05
*In vitro* drug release (%)	96.6 ± 0.74	96.0 ± 0.12	96.0 ± 0.02	96.7 ± 0.02	96.8 ± 0.61	95.6 ± 0.70
